# Significantly Suppressed Dielectric Loss and Enhanced Breakdown Strength in Core@Shell Structured Ni@TiO_2_/PVDF Composites

**DOI:** 10.3390/nano13010211

**Published:** 2023-01-03

**Authors:** Juanjuan Zhou, Wenying Zhou, Mengxue Yuan, Xinbo Dong, Jiebing Zhang, Xuejiao Zhang, Yanqing Zhang, Xiaolong Chen, Yanrong Chen, Xiangrong Liu

**Affiliations:** 1 School of Chemistry and Chemical Engineering, Xi’an University of Science & Technology, Xi’an 710054, China; 2 Department of Materials Science and Engineering, Pennsylvania State University, University Park, PA 16802, USA; 3 Department of Pharmacy, Xi’an Medical University, Xi’an 710021, China

**Keywords:** dielectric properties, metal, core–shell structure, polymer composites

## Abstract

An insulating shell on the surface of conductive particles is vital for restraining the dielectric loss and leakage current of polymer composites. So as to inhibit the enormous loss and conductivity of pristine nickel (Ni)/poly(vinylidene fluoride)(PVDF) composites but still harvest a high dielectric permittivity (*ε*_r_) when filler loading approaches or exceeds the percolation threshold (*f*_c_), pristine Ni particles were covered by a layer of titanium dioxide (TiO_2_) shell via a sol–gel approach, and then they were composited with PVDF. The impacts of the TiO_2_ coating on the dielectric performances of the Ni/PVDF composites were explored as a function of the filler concentration, the shell thickness and frequency. In addition, the dielectric performances were fitted using the Havriliak–Negami (H–N) equation in order to further understand the TiO_2_ shell’s effect on polarization mechanism in the composites. The Ni@TiO_2_/PVDF composites exhibit high *ε*_r_ and enhanced breakdown strength (*E*_b_) but remarkably suppressed loss and conductivity when compared with pristine Ni/PVDF because the TiO_2_ shell can efficiently stop the direct contact between Ni particles thereby suppressing the long–range electron transportation. Further, the dielectric performances can be effectively tuned through finely adjusting the TiO_2_ shell’ thickness. The resulting Ni@TiO_2_/PVDF composites with high *ε*_r_ and *E*_b_ but low loss show appealing applications in microelectronics and electrical fields.

## 1. Introduction

The rapid expansion of advanced power and electrical systems urgently need the robust, compact and highly efficient energy storage devices [1,2,3]. Compared with the extensively used electrolytic capacitors and supercapacitors, electrostatic capacitors play a vital role in much electrical power and electronic equipment [4], which are characterised by high operating electric voltage, fast charge–discharge rates and low energy loss [5,6,7,8]. The electrostatic capacitor highly depends on the dielectric materials with excellent electrical performances through electric polarization behavior to realize the charge storage and control subjecting to an applied electric field, which are widely used in pulse forming networks, medical defibrillators and power supplies, inter alia [9,10]. Compared with conventional dielectric materials used in electrostatic capacitors, polymers enjoy inherent advantages including high breakdown strength (*E*_b_), ultra–low dielectric loss, good self–healing behavior and excellent mechanical flexibility, but they also are confined to very low dielectric permittivity (*ε*_r_) [11,12,13]. Therefore, modification of polymers must be carried out to improve their dielectric performances [14].

To date, the high–ε_r_ polymer composites can be prepared generally by two extensively used approaches [15,16,17]. One is dispersing high–ε_r_ inorganic fillers into an insulating polymer [18], such as Pb(Zr_1−x_Ti_x_)O_3_ (PZT), BaSrTiO_3_, and others. Generally speaking, a decent increase in *ε*_r_ can be observed in the composites only when the filler concentration is nearly close to 65 wt% [19,20,21,22], which will inevitably destroy the processing [23,24,25,26,27,28], optical and mechanical performances of the composites [29]. Another approach to solve this question is based on percolating composites by using the conductive particles, such as copper (Cu) [23], zinc (Zn) [24], iron (Fe) [25], silver (Ag) [26] and carbon materials such as nanotubes (CNTs) [27]. The interpreting of those dielectric composites is always based on the micro–capacitor model and percolation theory [29]. When filler loading of conductive particles approaches the percolation threshold (*f*_c_), the *ε*_r_ will demonstrate a sharp increasing trend, which is always accompanied with giant undesirable dielectric loss, so, they cannot be regarded as the right materials for capacitor applications because of the unavoidably accompanied energy loss [30,31,32]. The conductive particles imposed a great challenge to precisely control the *f*_c_ composition to obtain desired dielectric performances in the products for industry applications [33]; hence, realizing the true balance between a high *ε*_r_ and very low loss or dissipation factor (*tanδ*) is still a huge problem in composites towards enhanced dielectric properties [34].

Hence, nowadays, a single strategy such as the above cannot concurrently satisfy the current development demands of the microelectronic industry where the excellent *ε*_r_, low loss as well as high *E*_b_ must be highly desired. A host of strategies have been reported to solve the above problem for conductive fillers/polymer system [35]. A prospective strategy to decrease the loss and reserve the relatively high *ε*_r_ is the use of an insulating interlayer between the conductive fillers and polymer matrix. The external shell can effectively stop the direct contact between the adjacent conductive particles, which markedly decreases the dielectric loss and leakage current. By reasonable designing, the *tanδ* of composites can be remarkably suppressed at low levels with choosing applicable interlayer materials [36]. The shells or interlayers have various forms, such as oxide (silicon dioxide (SiO_2_) [11], aluminum oxide (Al_2_O_3_) [26], nickel oxide (NiO) [19], aluminum nitride (AlN) [31], insulating polymers such as polystyrene, poly (vinyl pyrrolidone) and polyhedral oligomeric silsesquioxane), amongst others.

As a kind of hopeful conductive filler for polymer–based composites, nickel (Ni) has an extensive application due to their outstanding electrical, magnetic performances compared with other conductive particles, such as Cu, Ag and gold (Au). Now, despite the giant permittivity, the Ni/polymer exhibits a large dielectric loss and leakage current when the filler concentration is approaching the *f*_c_. So, various shells were built on the surface of pristine Ni to constrain the loss and leakage current of the composites [36,37,38,39,40]. For example, Li et al. [37] prepared the Ni@NiO/polyvinylidene fluoride (PVDF) composites displaying the desired *ε*_r_ and remarkably suppressed *tanδ*, and the dielectric performances can be adjusted by adjusting the NiO shell’ thicknesses. Zhu et al. [35] prepared the Ni@BaTiO_3_/epoxy composites, and they presented a clear improvement of *ε*_r_ from 2855 to 6397 at 10 kHz with increasing the shell BaTiO_3_ from 1.2 to 4 vol.%, and very low *tanδ* less than 0.04 at 10 kHz.

In this study, PVDF was selected as the polymer matrix because of the relatively high *ε*_r_ (>10 at low frequency range) compared with poly (methyl methacrylate), polydimethylsiloxane and epoxy, etc. At the same time, titanium dioxide (TiO_2_), as a semiconductor with a wide band gap about 3.0~3.2 eV, possesses the benefits of relative ease of synthesis, benign nature, photocorrosion stability and relatively high *ε*_r_ depending on crystal structure. Hence, we aim to utilize a TiO_2_ shell to suppress the *tanδ* and electrical conductivity of raw Ni [40]. In addition, the existence of a layer of TiO_2_ shell is expected to efficiently stop the long–term charge carrier’s migration among adjacent Ni particles, thus remarkably reducing the loss and leakage current. So, first, we successfully prepared a layer of TiO_2_ shell by a sol–gel method on the surface of Ni particles, and the obtained Ni@TiO_2_ filler was doped into the PVDF. Then, the influences of the filler concentration and the TiO_2_ thickness on the dielectric performances of the Ni@TiO_2_/PVDF composite were discussed in detail. The consequences are expected to shed a light on comprehending the potential polarization mechanism and the relationship between the microstructure and dielectric performances of the composites [32,34,41].

## 2. Experimental

### 2.1. Materials

The PVDF FR903 was provided by 3F New Materials Co. (Shanghai, China) and applied as the polymer matrix. The dimethylformamide (DMF) was provided by Chemical Reagent Co. Tianjin China. Spherical Ni powder with a diameter about 1–3 μm was offered by Tuya Metal Material Co., Hebei China. The tetrabutyl titanate (C_16_H_36_O_4_Ti) and glacial acetic acid (CH_3_COOH) were offered by Aladdin Biochemical Technology Co., Ltd., Shanghai China.

### 2.2. Preparation of Samples

**Figure 1:**
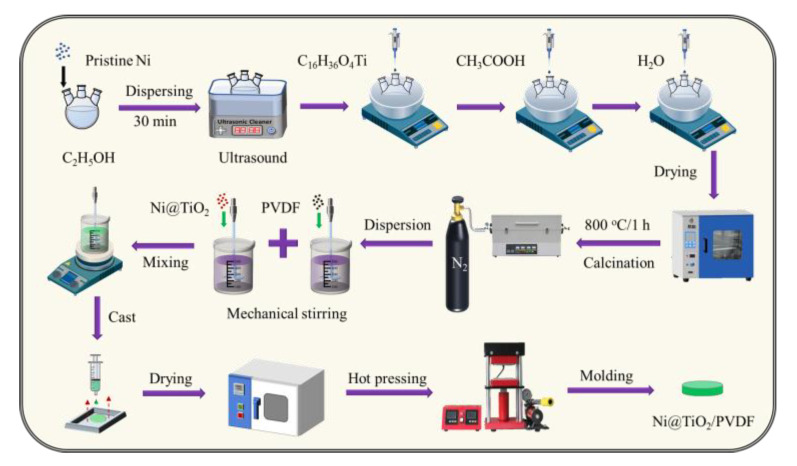
Schematic diagram of the preparation of Ni@TiO_2_ particles and corresponding Ni@TiO_2_/PVDF composites.

Figure 1 demonstrates the diagrammatical experimental process for the preparation of Ni@TiO_2_ particles and Ni@TiO_2_/PVDF composites. The core@shell structured Ni@TiO_2_ particles were prepared by a sol–gel method. First of all, 2.0 *g* Ni particles were uniformly dispersed in anhydrous ethanol (C_2_H_5_OH) at 30 °C. Next, the pH of the mixture was tuned to 4.0 via acetic acid under the rapid mechanical stirring. Then, 0.42 g of C_16_H_36_O_4_Ti and deionized water were dropwise added into the above mixture at 30 °C. and the reaction was lasted for 8 h. Subsequently, the resulting products were washed several times with anhydrous ethanol and deionized water. After that, the Ni@TiO_2_ precursors were acquired in oven at 90 °C for 8 h. In the end, the acquired products were heated in a tube furnace at 800 °C for 1 h under N_2_ atmosphere. The obtained products are labeled as Ni–1 (Ni@ 5 wt% TiO_2_), Ni–2 (Ni@ 10 wt% TiO_2_), Ni–3 (Ni@ 15 wt% TiO_2_), Ni–4 (Ni@ 20 wt% TiO_2_), respectively, and the Ni–0 represents the pristine Ni.

The Ni@TiO_2_/PVDF composites were prepared via a solution blending followed by a hot–pressing method. First, a certain mass of PVDF powders was disappeared in DMF solvent at 50 °C for 30 min by mechanical stirring, and a desired mass of core@shell structured Ni@TiO_2_ was uniformly mixed into DMF solution under the sonication for 30 min. Then, the above two suspensions were mixed by strong mechanical stirring for 6 h to acquire a uniform mixture which was further cast on a clear glass mold to evaporate the DMF solvent in a vacuum oven at 120 °C for 6 h. At the end, the sample was hot–pressed under a pressure of 10 MPa approximately at 190 °C for 15 min, to a thickness of about ~0.2 mm.

### 2.3. Characterizations

The chemical property of the Ni and Ni@TiO_2_ particles was tested by a Fourier transform infrared (FT–IR) spectrometer (Perkin–Elmer, Paragon1000, Waltham, Massachusetts, USA) in the wave range from 400 to 4000 cm^−1^. The phase composition of pristine Ni and various Ni@TiO_2_ particles was detected via a Shimadzu X-ray diffractometer–6000 (XRD) equipped with a graphite homochromatic instrument and a Cu anticathode (40 kV, 30 mA, scanning speed 2°/min). The outside elemental analysis of Ni@TiO_2_ particles was measured via X-ray photoelectron spectroscopy (XPS, Thermo Scientific K–Alpha, Thermo Fisher Scientific Co., Massachusetts USA).

The H–800 transmission electron microscope (TEM) from Hitachi Co. (Chiyoda Ward, Tokyo, Japan) was applied to examine the TiO_2_ outer layer structure on the outside of Ni particles. The JEOL JSM–6460LV (Japan Electronics Co., Xicheng, Beijing, China) scanning electron microscope (SEM) was applied to analyze the surface appearances of the Ni and Ni@TiO_2_ particles and the ruptured surface of the PVDF composites.

The dielectric performances of the Ni–0~Ni–4/PVDF composites were analyzed via an Agilent 4294 A impedance device at the frequency region from 40 to 10^7^ Hz. The *E*_b_ of all composites was tested by an electric breakdown tester (BDJC–50KV, Beiguangjing Instrument Equipment Co., LTD, Beijing, China). The *E*_b_ of the sample was placed between two copper ball electrodes and an alternating current (AC) voltage was loaded through a transformer (50 kV, 50 Hz). Both the electrodes and composites were immersed in an insulating oil preventing outside flashover and discharges. One electrode was earthed and the other electrode was applied to the raising AC voltage at a speed of 2 kV/s until the specimen failed, then the final breakdown voltage was recorded.

## 3. Results and Discussion

### 3.1. Characterizations of Core@Shell Ni@TiO_2_ Filler

**Figure 2:**
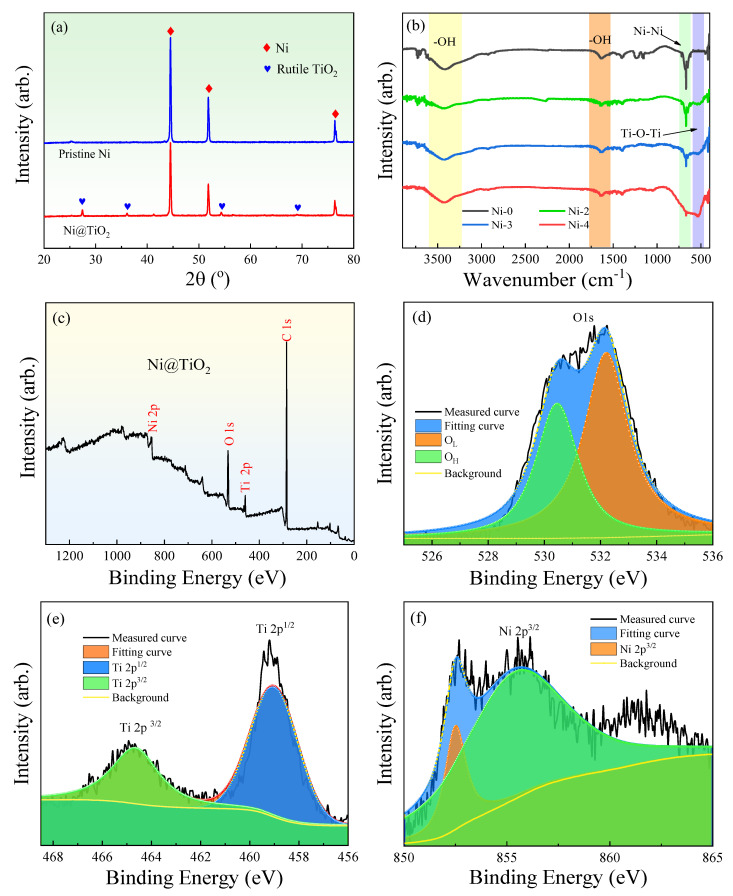
(**a**) XRD, (**b**) FT–IR, (**c**–**f**) XPS curves of Ni–0 and core@shell structured Ni@TiO_2_ particles.

Figure 2a demonstrates the XRD curves of pristine Ni and Ni@TiO_2_ particles. First, the XRD peaks in the curve of pristine Ni can well correspond to the lattice planes of Ni particles, for example, 44.5° (110), 51.8° (200) and 76.3° (220) [42]. Compared with the pristine Ni particles, the peaks intensity of Ni@TiO_2_ dramatically decline and several new peaks at 27.4° (110), 36.2° (101), 54.3° (211) and 69.1° (301) related to the lattice planes of TiO_2_ are observed [43]. Hence, the XRD analysis proves the formation of a TiO_2_ outer layer on the outside of the Ni core.

Figure 2b demonstrates the FT–IR curves of raw Ni particles and various Ni@TiO_2_ particles. The absorption peaks from 3400 to 3500 cm^−1^ and 1650 cm^−1^ can be linked to the vibration of –OH groups on the particles. Compared to the pristine Ni, the Ni@TiO_2_ particles possess a wide absorption peak at 580 cm^−1^ and the absorption peak intensity is increased with enhancing the shell thickness of TiO_2_, corresponding to the Ti–O stretching vibration peak. At the same time, the absorption peak of Ni@TiO_2_ at 680 cm^−1^ is significantly reduced when compared to the pristine Ni. Hence, the above results can indicate the successful coverage of a shell of TiO_2_ on the outside of Ni particles [44].

XPS was applied to analyze the elemental constitution and chemical status of the prepared Ni@TiO_2_ particles samples. The sample mainly includes Ni, Ti, and O elements, as indicated in Figure 2c, and the carbon emission peak can be found, which can be interpreted by the preparation approach and the transfer process of the sample into the UHV chamber. Figure 2d–f exhibits the XPS curves of element Ni, Ti, O separately in the Ni@TiO_2_ particle. The peaks appeared in 356.9 eV (Figure 2f) can be related to Ni 2p_3/2_ of the metallic Ni. As no peak is observed, consistent with Ni oxide variety in XRD and XPS, this demonstrates that the Ni core mainly exists as Ni^0^ in the core@shell structured Ni@TiO_2_ particles and the chemical state of Ni was not varied in the course of the reaction [44]. The sample demonstrates two Ti 2p_3/2_ (459.2 eV) and Ti 2p_1/2_ (464.9 eV) peaks as revealed in Figure 2e, which are ascribed to the Ti ^4+^ oxidation state based on measured XPS result. As the splitting of the 2p doublet was 5.7 eV, this binding energy also indicates the presence of TiO_2_ [44]. The XPS curve of O1s of the core@shell Ni@TiO_2_ particle (Figure 2d) can be fitted with the nonlinear least squares fitting program by Gaussian peak shapes. The first peak can be assigned to Ti–O of TiO_2_ (530.4 eV), and the second one is connected with the outside hydroxyl group (532.2 eV). Generally speaking, hydroxyl groups tested by XPS are attributed to the absorbed H_2_O. On account of the data of Figure 2c–f, we draw a conclusion that the Ni@TiO_2_ core@shell particles are really composed of TiO_2_ and Ni.

**Figure 3:**
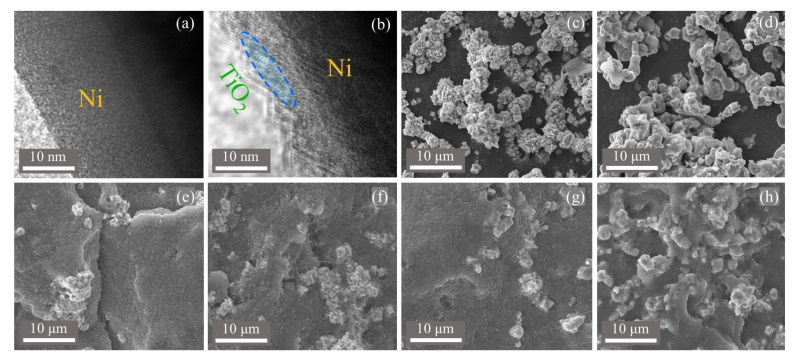
TEM and SEM pictures of (**a**,**c**) pristine Ni particles, (**b**,**d**) Ni@TiO_2_, (**e**) 30 wt% Ni–0/PVDF, (**f**) 50 wt% Ni–0/PVDF, (**g**) 30 wt% Ni–4/PVDF, (**h**) 50 wt% Ni–4/PVDF.

Figure 3 presents the TEM and SEM images of pristine Ni, Ni@TiO_2_ particles and their PVDF composites with different filler concentrations, separately. From Figure 3a, no obvious surface shell can be observed on the surface of the raw Ni, while, as shown in Figure 3b, a shell is clearly observed on the Ni core, thus giving the direct proofs for Ni@TiO_2_ particle. In addition, the shell displays the distinct shape of lattice fringes, which is the distinct evidence of crystalline TiO_2_, consistent with the results of XRD. Hence, TEM offers distinct evidence for the formation of crystalline core@shell structured Ni@TiO_2_ particles. From Figure 3c, the diameter of pristine Ni particles is 1–3 μm, and the Ni particles’ surface is smooth; but the Ni@TiO_2_ particles display a little rough surface due to the existence of a layer of TiO_2_ shell, as indicated in Figure 3d. From Figure 3e,f, the pristine Ni particles are not distributed well in the PVDF matrix, and certain Ni particles are found to crowd severely. While, from Figure 3g,h, the Ni@TiO_2_ particles are homogeneously dispersed in PVDF owing to the improved interfacial forces between the TiO_2_ shell and the PVDF matrix when compared to the Ni–0/PVDF system. As demonstrated in Figure 2b, after calcination, the –OH groups on the outside of TiO_2_ constitute the hydrogen bonds with the *F* atoms in PVDF matrix, thus, both the improved phase compatibility and interface interactions promote the homogeneous dispersion of fillers in the matrix, and play an important role in determining the dielectric performances of the composites. Hence, the above results collaboratively certify the successful preparation of core@shell structured Ni@TiO_2_ particles.

### 3.2. Dielectric Properties

**Figure 4:**
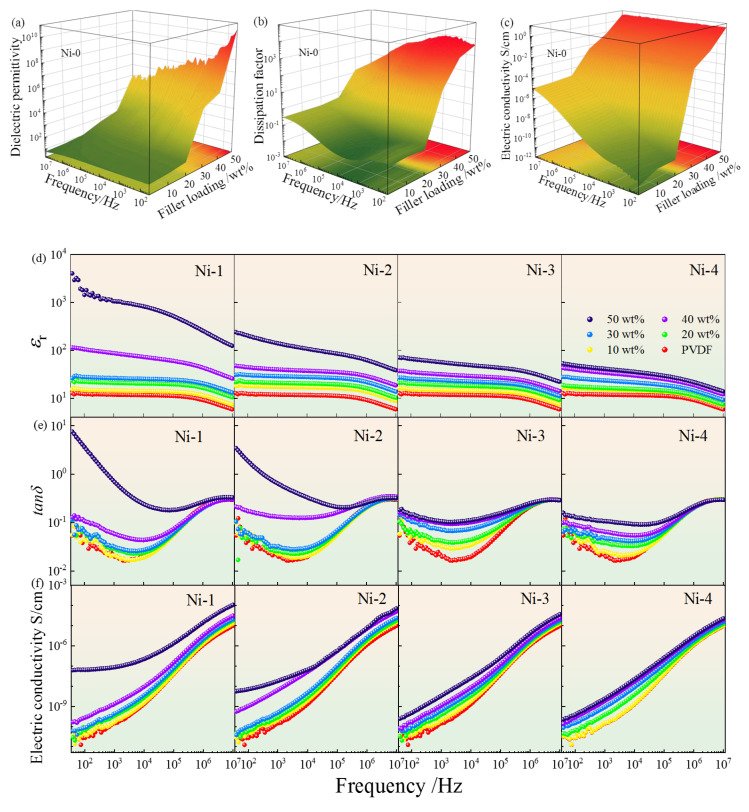
The frequency dependence of (**a**,**d**) *ε*_r_, (**b**,**e**) *tanδ* and (**c**,**f**) electric conductivity of Ni–0~Ni–4/PVDF composites containing various filler concentrations.

Figure 4 plots the dielectric properties of five composites with the filler concentrations and frequency [35]. All composites display the semblable dielectric behavior at the whole frequency range in Figure 4a,d. First, the *ε*_r_ of all composites demonstrate a clear drop with frequency from 40 to 10^7^ Hz owing to the relaxation polarization behavior, such as interface polarization (IP) at low frequency range and the dipole polarization hysteresis behavior at high frequencies. With enhancing the filler concentrations, all *ε*_r_ rise at the whole frequency range [37]. The specific causes will be discussed below corresponding to the various core@shell structured fillers [38].

As well known, the PVDF matrix possesses a relatively low *ε*_r_ usually less than 10. For Ni–0/PVDF composites [2,24,39], the *ε*_r_ is obviously enhanced by the incorporation of Ni–0 particles, especially at high filler concentrations. The change of *ε*_r_ can be split into two courses. At a filler concentration less than 20 wt%, the *ε*_r_ keeps at relatively low level, which can be interpreted by the insufficient numbers of charge carriers’ and micro capacitors formed in composites [40]. When the filler concentration exceeds 30 wt%, the *ε*_r_ demonstrates an exponential growth due to the continuously formed microcapacitors and enhanced IP effect. However, unfortunately, the *tanδ* of the Ni–0/PVDF composites exhibits an enormous surge in Figure 4b. For example, the *tanδ* of PVDF with 50 wt% Ni–0 particles reaches 8520 at 10^3^ Hz because a large number of conductive particles of Ni have connected with each other and formed a conductive network producing the tremendous leakage current [41].

For Ni–1~Ni–4/PVDF composites, the TiO_2_ can restrain the *ε*_r_ of Ni/PVDF due to the suppressed long–range electron transport, and the suppression effect enhances gradually, as indicated in Figure 4d. For example, *ε*_r_ of the composites with 50 wt% of Ni–0 is 1.07 × 10^7^ at 10^3^ Hz, corresponding to 1060, 141, 56.4 and 41.2 in the PVDF with Ni–1~Ni–4 at the same filler concentrations, severally. The gradually decreased permittivity in Ni@TiO_2_/PVDF systems can be explained as follow. The semi–conductor TiO_2_ with a wide forbidden band effectively prevents the charge carrier’s migration between pristine Ni particles, efficaciously promoting the formation of leakage current, and remarkably declining space charge polarization [42,43,44,45,46]. However, compared to PVDF, the Ni@TiO_2_/PVDF composites still exhibit much large permittivity.

**Figure 5:**
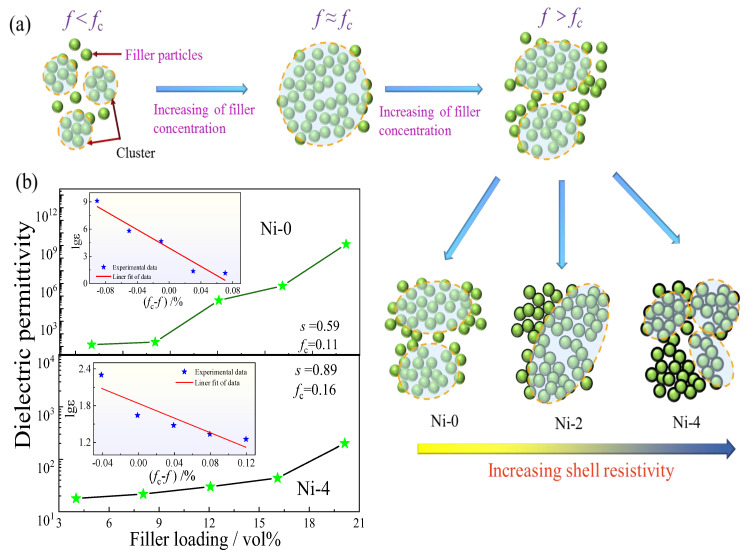
(**a**) Filler particles are clustered into polarizable domains (yellow blob), which describes the polarization region of polymer composites, (**b**) *ε*_r_ as a function of filler concentration at 10^2^ Hz for Ni–0/PVDF and Ni–4/PVDF composites, and the insert graph displays a log–log curves of *ε*_r_ as a function of *f*_c_ − *f*.

**Figure 6:**
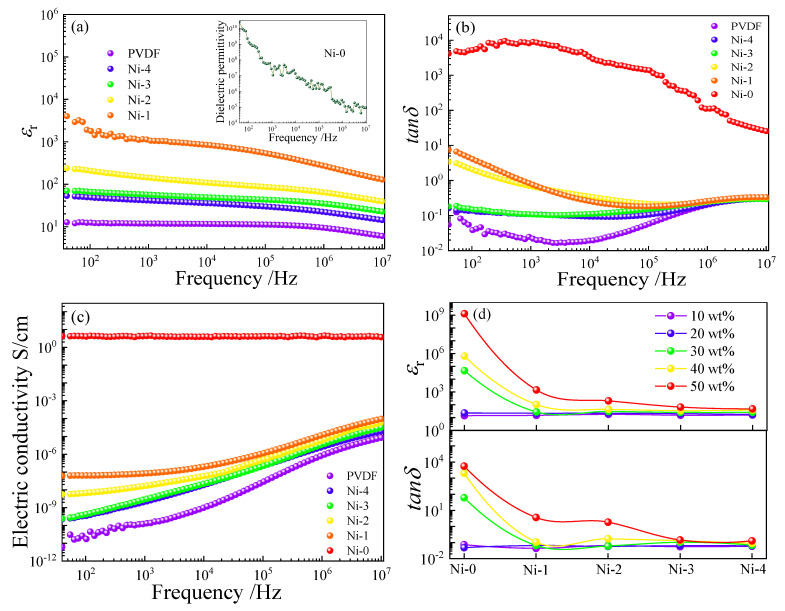
(**a**) *ε*_r_, (**b**) *tanδ* and (**c**) electric conductivity of Ni–0~Ni–4/PVDF composites with 50 wt%, the inlet standing for the Ni–0/PVDF composites, (**d**) variations of *ε*_r_ and *tanδ* of the composites containing various fillers at different concentrations (at 10^2^ Hz).

It is worth noting that the *ε*_r_ of Ni–0~Ni–4/PVDF composites exhibits a tremendous change with the filler concentrations from 10 wt% to 50 wt%, which can be explained by the equation:

(1) $$\varepsilon =\varepsilon_{m}{\left(f_{c}-f\right)}^{-s}\;\;\;\;\;\;\;\;\;\;\;\;for\;f\;<\;f_{c}$$

where $\varepsilon_{m}$ represents the *ε*_r_ of the PVDF matrix, *s* stands for a considerable index factor close to 1. The log–log patterns of *ε*_r_ and *f* are displayed in the insert pattern in Figure 5b, and the greatest linear fitting of *ε*_r_ demonstrates that the *f*_c_ of Ni–0/PVDF and Ni–4/PVDF composites are 11.0 vol% and 16.0 vol%, severally, and *s* is close to 0.59 and 0.89. In addition, Figure 5a displays the principle scheme of the cluster polarization mechanism. In percolative composites, based on the conductive Ni particles distance, the Ni particles could be divided into diverse polarizable clusters (yellow circle). The Ni particles demonstrate a closer connection when they belong to the same cluster rather than belonging to different clusters [47,48,49,50]. When an e–field is applied, the electrons can transport a whole cluster region completely, and electrons migration can be limited to the within filler clusters, even under high frequency, due to the distance of adjacent particles in the same cluster spacing is far less than in the other clusters, as ascribed in Figure 5a [2]. The above mentioned electron transport can be regarded as one of dipolar orientation in polar materials, which is the basics of polarization in percolative composites (indicated as cluster polarization). According to the cluster polarization mechanism, the large clusters will possess a large number of electrons and electron displacement, resulting in the increased *ε*_r_ in Figure 6a, and will spend a longer time to accomplish the long–range electron migration, which could explain the increase of *tanδ* in the measured frequency range. The *ε*_r_ demonstrates a diverge tendency (as ascribed in Figure 6a) when the filler concentrations go across the *f*_c_, which can be interpreted by the divergence of cluster size in typical percolative composites in Figure 5a. It is obvious that the change of *ε*_r_ with raising the filler concentration is very alike to the increasing shell thickness of TiO_2_ from Ni–1 to Ni–4, both of which radically suppress electron migration in adjacent filler particles. Hence, for percolated composites, increasing shell thickness of TiO_2_ will raise the electrical resistivity of filler–shells and suppress the interparticle electron transport, which is the same effect as decreasing filler loading, which arouses the change of filler cluster size and *ε*_r_ in Figure 5a, corresponding to the increasing *f*_c_ [50]. On account of the cluster polarization mechanism, a hopeful strategy to gain a high *ε*_r_ but low *tanδ* in percolative composites is to use the suited filler loadings and adjust the right thickness of TiO_2_ shell, until an increased filler cluster size is acquired (high *ε*_r_) and long–range electron migration is restrained (low loss).

Generally speaking, the dielectric loss can be deemed to the aggregation of electric leakage loss and polarization loss in the whole frequency region. The *tanδ* of Ni–0~Ni–4/PVDF composites are depicted in Figure 4b,e. All composites display a first reduce and second increase trend at whole frequency range. The low frequency dielectric behavior can be connected to the IP and electric leakage. The IP behavior primarily comes from the charge carrier traps at the interfaces between diverse phases, which possess significant difference in dielectric performances, and the electric leakage plays a dominant role in the Ni–0/PVDF composites. Conversely, the high–frequency dielectric behavior is a classic Debye relaxation behavior because of the C–F dipoles orientation polarization relaxation in the PVDF matrix [51,52].

From Figure 4b, the change in *tanδ* for the Ni–0/PVDF composites exhibits no dependance of the filler concentration at *f* < *f*_c_, where the adjacent Ni particles are mainly isolated far away; however, when the filler concentration is close to the *f*_c_, the *tanδ* first increases by an exponential form, and then decreases sharply with frequency. For example, the PVDF with 50 wt% Ni–0 demonstrates an enormous *tanδ* = 8520 at 10^3^ Hz compared to only 0.026 for composite with 20 wt% filler. The tremendous *tanδ* at low frequency range can be interpreted by the direct current (DC) conduction from the formation of conductive network of Ni–0 through the composites, hence, resulting in the amassed charge carriers at the different phases interface and electron conduction process. In addition, the *tanδ* declines over the whole frequency region at the uniform filler concentration, due to the fact that the charges migration cannot keep place with the transformation of external electric field, resulting in the lack of electronic oscillations. Therefore, the enormous *tanδ* can be found in the Ni–0/PVDF composites with intense frequency dependence.

For the PVDF filled with various types of Ni@TiO_2_ displayed in Figure 4e, all composites demonstrate much lower *tanδ* than that of Ni–0/PVDF at the frequency from 40 to 10^5^ Hz, and almost no percolation behavior can be found when the filler concentration is more than *f*_c_. Moreover, the *tanδ* decreases with the piecemeal increase in thickness of TiO_2_ shell [34]. The dramatically restrained *tanδ* is due to the TiO_2_ shell, and it works as an interlayer between Ni cores stopping them from directly touching. Hence, the long–range transport of free charge carriers can be obviously restrained, markedly decreasing the leakage current. However, for the Ni–0/PVDF systems, the overwhelming majority of Ni particles in the PVDF matrix will start touching and overlapping with each other, and it will accelerate the formation of conductive paths, resulting in a heavy electron conduction process. Hence, as exhibited in Figure 6b, the conduction loss of Ni–1~Ni–4/PVDF composites can be prominently restrained to a much lower level. For instance, the *tanδ* (10^2^ Hz) of PVDF with 50 wt% of Ni–1, Ni–2, Ni–3 and Ni–4 are 3.63, 1.85, 0.164 and 0.129, separately, in comparison with 300 for the Ni–0/PVDF under the uniform filler concentration.

For a composite, dielectric loss (*ε*′′) can be expressed by the equation [10,23]:

(2) $$\varepsilon^{\prime \prime }=\varepsilon_{dc}^{\prime \prime }+\varepsilon_{MW}^{\prime \prime }+\varepsilon_{D}^{\prime \prime }$$

where, $\varepsilon_{dc}^{\prime \prime }$ represents the conduction loss, $\varepsilon_{MW}^{\prime \prime }$ is regarded as loss originated from IP, and $\varepsilon_{D}^{\prime \prime }$ is on behalf of the dipole loss in the matrix [23].

The conduction loss is represented as Equation (3) [37,39]:

(3) $$\varepsilon_{dc}^{\prime \prime }=\frac{\sigma_{dc}}{2\pi f}$$

The $\sigma_{dc}$ and f represent DC conductivity and frequency, severally.

According to the Equations (2) and (3), the conduction loss plays a minor role in Ni–1~Ni–4/PVDF composites, because the leakage current can be restrained by the semi–conductor TiO_2_ interlayer. Therefore, the introduction of the TiO_2_ shell in the Ni/PVDF composites is beneficial to whittle the conductive loss at low frequency range [36].

Figure 4c,f show the dependence of the alternating current (AC) conductivity on frequency and filler concentration for the Ni–0~Ni–4/PVDF composites. Significantly, the AC conductivity of all samples exhibits a continuous increase tendency with enhancing the filler concentrations and frequency. When the Ni–0 concentration is less than the *f*_c_ (about 11.0 vol%), the AC conductivities slightly increase with the filler concentration, which can be put down to the even dispersion of insufficient numbers of Ni–0 in PVDF matrix. When the Ni–0 concentration is more than *f*_c_, the Ni–0 particles connect one another and form the conductive network in the PVDF matrix, which results in the transformation from insulator to conductor, and the direct current (DC) conduction takes place in the Ni–0/PVDF which is independent of frequency and increases with the filler loading in Figure 4c. The connection between contiguous Ni–0 particles will play an important role in the AC conductivity for the whole frequency region, resulting in enormous leakage currents and bringing a tremendous dielectric loss, which will destroy the electrical resistivity of the Ni–0/PVDF composites.

As depicted in Figure 4f, the AC conductivity of the Ni–1~Ni–4/PVDF composites in the measured frequency region is remarkably restrained in spite of the filler concentrations at a high level, attributed to the TiO_2_ interlayer which efficaciously suppresses the formation of conductive network by stopping the adjacent Ni–0 direct connection with each other. In addition, a remarkable suppression AC conductivity can be found under the same filler concentrations due to the increasing inhibition on electron conduction process and transport of free charge carriers with the increasing thickness of TiO_2_ interlayer, which works as an efficacious barrier, and significantly restrains the AC conductivity, thus resulting in the decrease of loss [53]. It should be noted that the TiO_2_ interlayer not only remarkably decreases the *tanδ*, electric conductivity but also simultaneously reserves a relatively high *ε*_r_. in the composites. Furthermore, the dielectric performances of Ni@TiO_2_/PVDF can be tuned by choosing suitable TiO_2_ shell thickness.

In order to further understand the polarization mechanism in the PVDF with core@shell structured Ni@TiO_2_ with diverse shell thicknesses, we must isolate single polarization from the diverse polarizations. The imaginary part of permittivity (*ε*′′) vs. real part of permittivity (*ε*′) are concurrently fitted by the Havriliak–Negami (H–N) equation described as follows [15]:

(4) $$\varepsilon^{*}\left(\omega \right)=\varepsilon_{\infty }+\underset{i}{\sum }\left[\frac{\Delta \varepsilon_{i}}{{\left(1+{\left(j\omega \tau_{HNi}\right)}^{\alpha_{i}}\right)}^{\beta_{i}}}\right]-j\frac{\sigma_{dc}}{\varepsilon_{0}\omega }$$

where the $\tau_{HNi}\;$ is on behalf of the relaxation time and $\Delta \varepsilon_{i}$ is regarded as the dielectric relaxation strength; $\alpha_{i}$ and $\beta_{i}$ represent the symmetrical and asymmetrical broadening of the distribution of relaxation times (0 <$\;\alpha_{i}$; $\alpha_{i}\beta_{i}\;$< 1), respectively; $\sigma_{dc}$ and $\varepsilon_{0}$ stand for the conductivity loss and *ε*′ in free space, respectively, and *i* is the different relaxations in the dielectric composites.

**Figure 7:**
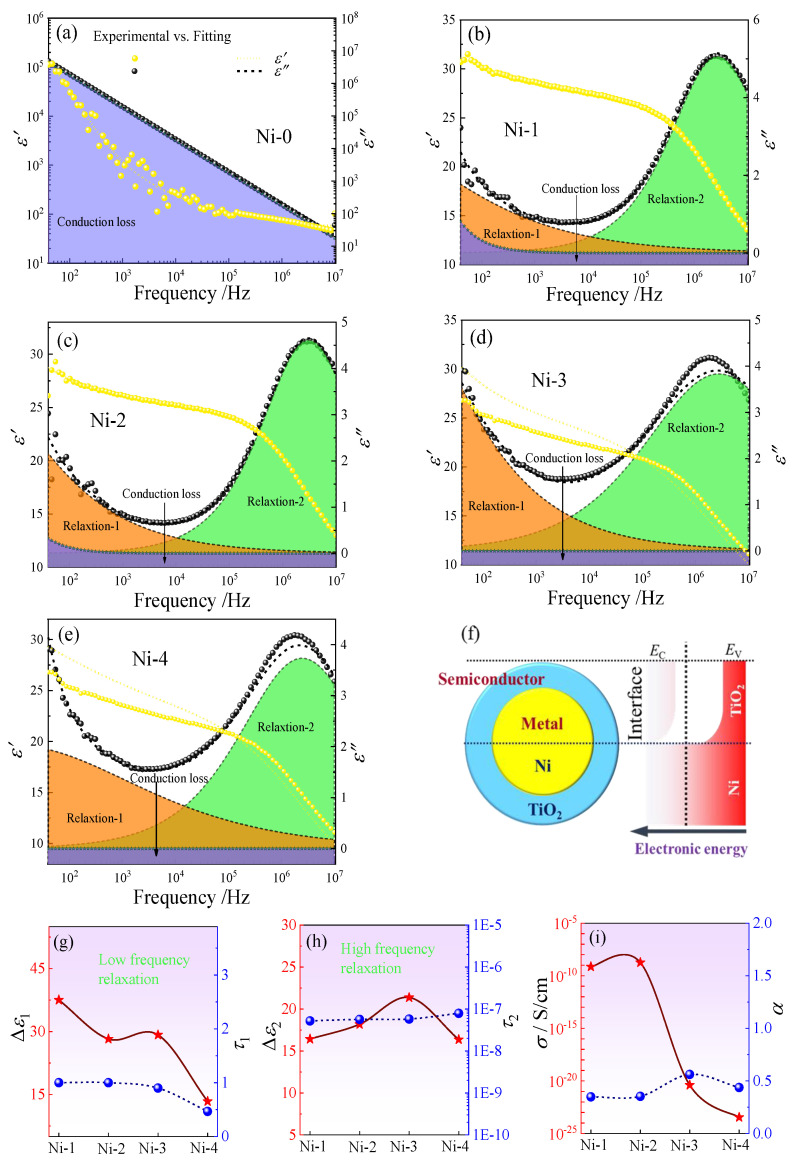
(**a**–**e**) Dielectric spectra of PVDF composites including various fillers (Ni–0~Ni–4) at 30 wt% deconvolutions into separate relaxations by H–N equation, (**f**) the schematic diagram of the influence of energy gap of core@shell structured Ni@TiO_2_, (**g**–**i**) summary and evolution of best–fitting parameters with different shell thicknesses.

**Figure 8:**
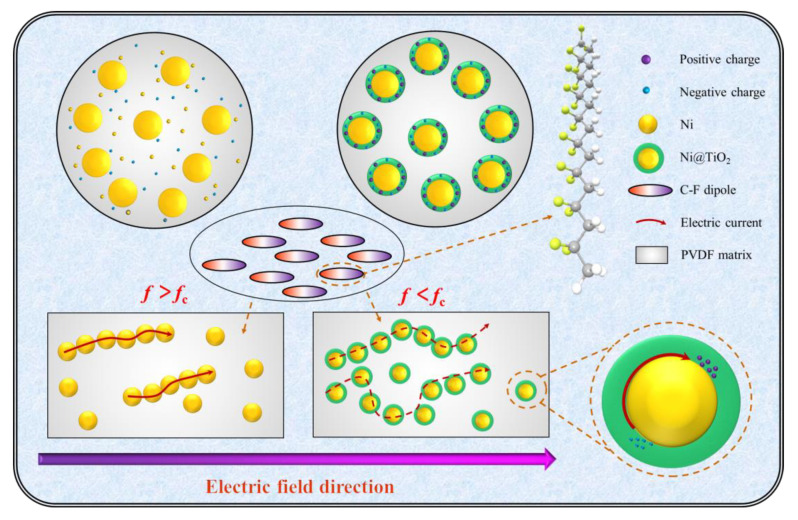
The brief diagrammatic drawing of the conductive network and the influence of the TiO_2_ interlayer on dielectric performances of composites.

The deconvolution of PVDF containing the Ni–0~Ni–4 particles at 30 wt% are displayed in Figure 7a–e. For Ni–0/PVDF composites, the conduction loss plays an important role in dielectric loss which results in the contribution to different relaxations not being clearly divided in Figure 7a, due to the formed conductive network from Ni–0 particles. For Ni–1~Ni–4/PVDF composites, the dielectric behavior can be described via two different dielectric relaxation peaks in Figure 7b–e. The polarization relaxation peak occurring at high frequency range (about 10^6^~10^7^ Hz) is related to the segmental relaxation in PVDF amorphous molecular chains, and has been researched widely, which will not be discussed minutely in this paper, as described in relaxation–2 in Figure 7b–e. The polarization relaxation peak stimulated at low frequency range (about 40~10^4^ Hz) is the reason of the remarkable improvement of *ε*_r_ in Ni@TiO_2_/PVDF composites, which is caused by the Ni@TiO_2_ fillers’ contribution, as described in relaxation–1 in Figure 7b–e. On the one hand, the polarization relaxation peak of Ni@TiO_2_ particles can be explained by the electron displacement and the free electron will accumulate at the interlayer of TiO_2_ when an external electric field is used, on account of unsymmetrical distribution of charge carriers which serve as permanent electric dipoles and intensify the polarization behavior of core@shell particles, resulting in the remarkable improvement of *ε*_r_ in Ni@TiO_2_/PVDF composites. On the other hand, the conductive Ni core easily acquires sufficient energy and the electron can lightly jump to the conduction band (CB) and form the conductive network, because the valence band (VB) and CB are overlapping and the band gap is equal to 0. Meanwhile, for core@shell structured Ni@TiO_2_, the TiO_2_ possesses a wide band gap between CB and VB up to 3.0 eV, which can stop the electronics tunneling and jumping between the adjacent Ni particles, as indicated in Figure 7f, and make electrons migrate only inside the conductor core, thereby inhibiting the formation of conductive network (Figure 8) and resulting in the decreasing of conductive loss. Apart from the two relaxations mentioned above, the quasi–DC conductivity loss plays an essential role in dielectric loss and it increases observably at low frequency range, arising from the surplus charge’s long–range transport, such as impurities and chemical leftovers. In addition, electron displacement and charge migration are strongly associated with the IP behavior, which plays a vital role in the dielectric properties [54].

Figure 7g–i give the best–fitting results. The relaxation peak intensity in high–frequency (Δ*ε*_2_) is 20 and the relaxation time (*τ*_2_) is 10^−8^ s, approximately, which displays a tiny change with the various shell thicknesses of TiO_2_ in Ni–1~Ni–4/PVDF composites in Figure 7h. It is easy to understand that the relaxation mentioned above derives from the PVDF matrix, which occupies the same concentrations in the discussed composites. The relaxation peak intensity in low–frequency (Δ*ε*_1_) is obviously linked with the filler polarization, which is not similar to the relaxation behavior of PVDF matrix, displaying the non–linear correlation with the change of the shell thickness of TiO_2_ in Ni–1~Ni–4/PVDF composites in Figure 7g. The relaxation peak strength and relaxation time demonstrate a continuously decrease trend with increasing of shell thickness of TiO_2_ in the Ni–1~Ni–4/PVDF composites. Clearly, the decrease of low–frequency relaxation (relaxation–1) from Ni–1/PVDF to Ni–4/PVDF composites is also proved in Figure 4 [48].

In addition, the shape parameters (*α*) of all relaxations demonstrate a tiny change with different shell thicknesses of TiO_2_ in Ni–1~Ni–4/PVDF composites, suggesting a semblable polarization mechanism in Figure 7i [48]. At the same time, the electric conductivity (*σ*_dc_) reveals an everlasting decrease with increasing the shell thickness of TiO_2_ in Ni–1~Ni–4/PVDF composites, corresponding to the long–term electron’s migration, stopped effectively by the TiO_2_ shell in Figure 7i.

**Figure 9:**
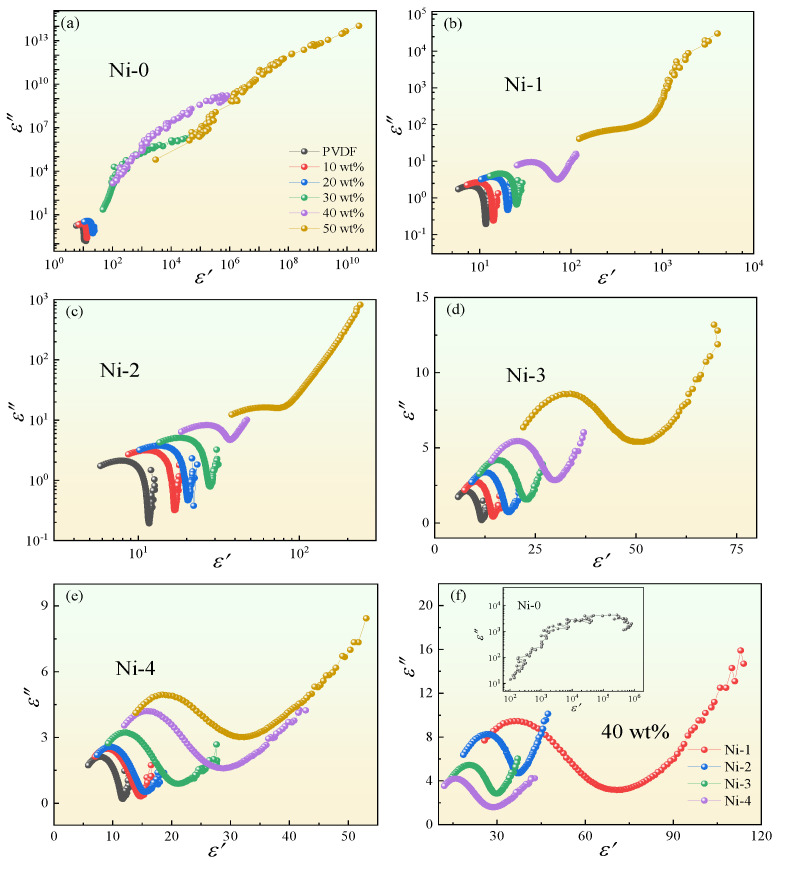
Cole–Cole curves of ε′′ vs. ε′ for composites containing Ni–0~Ni–4 particles (**a**–**e**) containing different filler concentrations, and (**f**) ε′′ vs. ε′ for PVDF including diverse fillers at 40 wt%. The insert is for the pristine Ni/PVDF.

Figure 9 display the Cole–Cole graphs of *ε*′′ vs. *ε*′ for PVDF including pristine Ni and a variety of Ni@TiO_2_ particles. It is fairly obvious that the Cole–Cole graphs of all composites reveal the semblable features, i.e., two similar semicircular arcs. The first small semicircular arc represents the fast relaxation corresponding to the C–F dipoles polarization behavior in PVDF matrix, occurring at high frequency region; in addition, the second large semicircular arc is on behalf of the IP effect, appearing at low frequency region. Figure 9a–e clearly demonstrates the loss from conductivity increases with the filler concentration, and the extent of deviation of the large semicircle from the *ε′*–axis also reflects the conductivity loss extent. Generally speaking, the polymer composites cannot be interpreted by the monochromatic dispersion mechanism, therefore the Cole–Cole model is usually used to interpret the complex dielectric behaviors. The Cole–Cole equation is made up of the real part and imaginary part of *ε*^∗^, and it can be expressed as follows:

(5) $$\varepsilon^{*}\left(\omega \right)=\varepsilon^{\prime }\left(\omega \right)-i\varepsilon^{\prime \prime }\left(\omega \right)=\varepsilon_{\infty }+\frac{\varepsilon_{s}-\varepsilon_{\infty }}{1+i\omega \tau }-i\frac{\sigma }{\omega }$$

(6) $$\;tan\delta =\frac{\;\varepsilon \;\prime \prime }{\;\varepsilon \;\prime }$$

where σ is the electric conductivity, $\varepsilon_{\infty }$ is on behalf of the optical $\varepsilon^{\prime }\left(\omega \to \infty \right)$, $\varepsilon_{s}$ is the electrostatic $\varepsilon^{\prime }\left(\omega \to 0\right)$, ω=2πf is regarded as the angular frequency, and τ is relaxation time [7,14].

In addition, $i\frac{\sigma }{\omega }$ in Equation (5) represents the contribution from electric conductivity, which significantly affects the large semicircle’ shape of Cole–Cole splines. As the filler concentration increases, the electric conductivity rises sharply in Figure 9a–e, and the DC conductivity can significantly influence the curve’ form, and it diverges from semicircle and demonstrates a distinctly distorted arc–shape. With the filler loading increasing from 10 wt% to 50 wt%, from the Ni–0/PVDF to the Ni–1~Ni–4/PVDF composites, the semicircle’ diameter of Cole–Cole curves does not rise significantly, relating to the rising trend of electric conductivity. Compared to the Ni–0/PVDF, the Ni–1~Ni–4/PVDF composites possess a continuous increase in the shell thickness, which results in a significant enhancement of electrical resistivity as ascribed in Figure 5a and suppresses the ε′′ relaxation behavior, and the smaller semicircle and relaxation curve at low frequency region can be observed. For Figure 9f, by contrasting the four kinds of Ni@TiO_2_/PVDF composites at 40 wt%, severally, the large semicircle displays a remarkable decrease in the diameter of the semicircle, corresponding to the decline of electric conductivity in Figure 4f.

**Figure 10:**
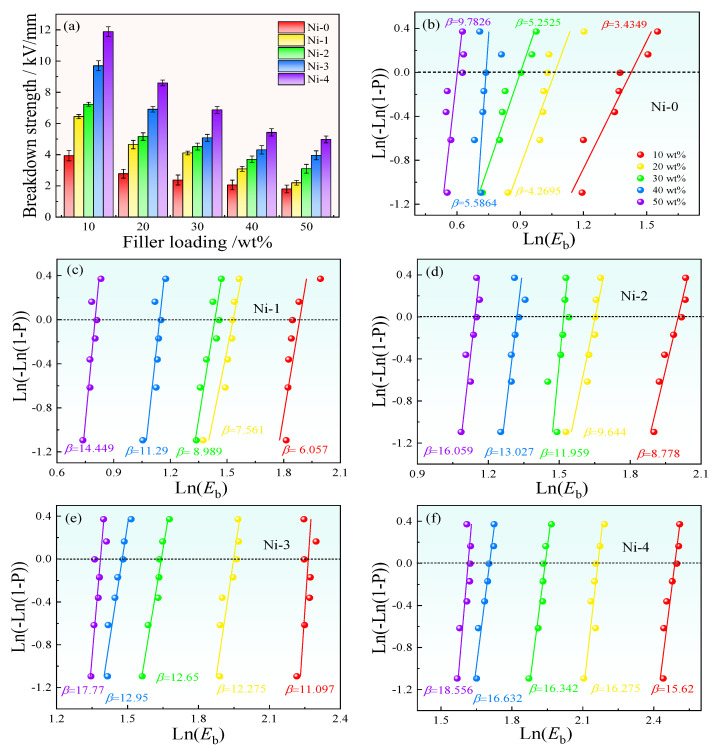
(**a**) The E_b_ changes with various filler concentrations for five composites, (**b**–**f**) the Weibull distribution for Ni–0~Ni–4/PVDF composites.

Despite the Ni–1~Ni–4/PVDF composites possessing a relatively splendid dielectric performance under the low voltage, another critical factor is improving the high–field E_b_ of dielectric materials. Hence, Figure 10 represents the E_b_ and the typical Weibull distributions towards Ni–0~Ni–4/PVDF composites [49]. For Figure 10a, it is clear that the E_b_ for all composites display a decline tendency with the increase of filler concentrations. The decline in E_b_ can be interpreted by the imparity of ε_r_ and electric conductivity between the PVDF and the fillers. With increasing filler concentrations will generate a remarkable concentration and distortion in partial electric field in the composites [50]. Furthermore, with enhancing of the TiO_2_ shell thickness, the E_b_ of the composites demonstrates a significant increasing tendency. For instance, under the uniform filler concentration at 50 wt%, the E_b_ of Ni–0~Ni–4/PVDF are 2.78, 4.65, 5.16, 6.91 and 8.6 kV/mm, respectively [35]. The results suggest that the semi–conductor TiO_2_ shell possesses a suitable ε_r_ and electric conductivity, and works as an effective buffer layer, which lessens the difference in conductivity and ε_r_ between PVDF matrix and Ni fillers, and effectively mitigates the partial electric field concentration and distortion [10].

In addition, the E_b_ can be analyzed by a traditional two–parameter Weibull distribution equation, which is demonstrated as follows, and the results are revealed in Figure 10b–f:

(7) $$P=1-exp\left[-{\left(\frac{E_{b}}{\alpha }\right)}^{\beta }\right]$$

where *P* is behalf of the cumulative probability of electrical failure, *β* represents the rate of curves, which is quantified the distribution of the experimental results, and *α* stands for the *E*_b_ at a cumulative failure probability of 0.632.

The linear fitting results and Weibull parameters are displayed in Figure 10b–f [46,48]. The linearity of all lines is fitting for the distribution data point of the Weibull equation, and the parameters are remarkably exceeding 1, suggesting the possible distribution of these *E*_b_ value corresponding to the two–parameter Weibull model [53,54,55].

## 4. Conclusions

In order to effectually restrain the large *tanδ* of pristine Ni/PVDF, core@shell structured Ni@TiO_2_ particles were prepared by a moderate sol–gel strategy. The XRD, FT–IR, XPS, SEM and TEM measurements prove that a semi–conductor TiO_2_ shell was encapsulated on the surface of Ni cores. The *ε*_r_ of Ni@TiO_2_/PVDF composites declines under the identical filler concentrations, but the *tanδ* and electric conductivity are strongly restrained to very low levels, compared to the Ni/PVDF composites. The suppressing effect of *tanδ* and electric conductivity can be interpreted by the TiO_2_ shell on the surface of Ni core, which stops the conductive Ni particles from directly touching each other, hence, reducing the long–range electron transport and leading to the low loss and electrical conductivity. A clear higher *f*_c_ is observed in Ni@TiO_2_/PVDF composites also attributed to the TiO_2_ which restrains the *tanδ* and suppresses electric conductivity. Additionally, the Ni@TiO_2_/PVDF composites possess a higher *E*_b_ as comparison with Ni/PVDF due to the interlayer TiO_2_ working as buffer layer relieving the electric field distortion. Moreover, the fitting results from the Cole–Cole and Havriliak–Negami (H–N) equations based on experimental data theoretically support the drawn conclusion and confirm the TiO_2_ shell’s influence on dielectric performances of the composites. The prepared Ni@TiO_2_/PVDF composites with relatively high *ε*_r_, low *tanδ*, and improved *E*_b_, have appealing application prospects in the electrical industry.

## Data Availability

The data presented in this study are available on request from the corresponding author.
